# Utility of 7 Tesla Magnetic Resonance Imaging in Patients With Epilepsy: A Systematic Review and Meta-Analysis

**DOI:** 10.3389/fneur.2021.621936

**Published:** 2021-03-19

**Authors:** Ji Eun Park, E-Nae Cheong, Da Eun Jung, Woo Hyun Shim, Ji Sung Lee

**Affiliations:** ^1^Department of Radiology and Research Institute of Radiology, Asan Medical Center, University of Ulsan College of Medicine, Seoul, South Korea; ^2^Department of Medical Science and Asan Medical Institute of Convergence Science and Technology, Asan Medical Center, University of Ulsan College of Medicine, Seoul, South Korea; ^3^Department of Pediatrics, Ajou University School of Medicine, Suwon, South Korea; ^4^Department of Statistics, College of Medicine, Ulsan University, Asan Medical Center, Seoul, South Korea

**Keywords:** epilepsy, 7 Tesla, ultra-high field, magnetic resonance imaging, systematic review

## Abstract

**Objective:** 7 Tesla magnetic resonance imaging (MRI) enables high resolution imaging and potentially improves the detection of morphologic abnormalities in patients with epilepsy. However, its added value compared with conventional 1.5T and 3.0T MRI is unclear. We reviewed the evidence for the use of 7 Tesla MRI in patients with epilepsy and compared the detection rate of focal lesions with clinical MRI.

**Methods:** Clinical retrospective case studies were identified using the indexed text terms “epilepsy” AND “magnetic resonance imaging” OR “MR imaging” AND “7T” OR “7 Tesla” OR “7T” in Medline (2002-September 1, 2020) and Embase (1999-September 1, 2020). The study setting, MRI protocols, qualitative, and quantitative assessment were systematically reviewed. The detection rate of morphologic abnormalities on MRI was reported in each study in which surgery was used as the reference standard. Meta-analyses were performed using a univariate random-effects model in diagnostic performance studies with patients that underwent both 7T MRI and conventional MRI.

**Results:** Twenty-five articles were included (467 patients and 167 healthy controls) consisting of 10 case studies, 10 case-control studies, 4 case series, and 1 cohort study. All studies included focal epilepsy; 12 studies (12/25, 48%) specified the disease etiology and 4 studies reported focal but non-lesional (MRI-negative on 1.5/3.0T) epilepsy. 7T MRI showed superior detection and delineation of morphologic abnormalities in all studies. In nine comparative studies, 7T MRI had a superior detection rate of 65% compared with the 22% detection rate of 1.5T or 3.0T.

**Significance:** 7T MRI is useful for delineating morphologic abnormalities with a higher detection rate compared with conventional clinical MRI. Most studies were conducted using a case series or case study; therefore, a cohort study design with clinical outcomes is necessary.

**Classification of Evidence:** Class IV Criteria for Rating Diagnostic Accuracy Studies.

## Key Points:

- In a systematic review, 7T MRI showed superior detection and delineation of morphologic details compared with conventional 1.5T or 3.0T MRI.
- 7T showed a superior detection rate of 65% compared with a 22% detection rate for 1.5T or 3.0T MRI.
- A cohort study in patients with epilepsy is necessary to evaluate the diagnostic performance of 7T MRI.

## Introduction

Approximately 20–40% of individuals with epilepsy do not respond to anti-seizure drug therapy (1). The most effective operative management involves a focal cortical resection with excision of the epileptogenic cortex and analysis of the underlying pathologic findings (2). Chronic focal epilepsy includes mesial temporal sclerosis (MTS), focal cortical dysplasia, neoplastic lesions, cavernous hemangioma, remote cerebral infarction, and posttraumatic encephalomalacia (3). In cases of focal lesional epilepsy, resective surgery is the most effective treatment that allows patients to become seizure-free, improving their quality of life (4).

However, 20–30% of patients with focal epilepsy are “MRI-negative” on clinical MRI, meaning that they do not have an identifiable lesion on 1.5T or 3.0T MRI (5, 6). Advances in imaging techniques by 7 Tesla MRI might improve the visualization of smaller anatomical structures and allow detailed pathological findings with a high spatial resolution by reducing the voxel size related to the increased signal-to-noise (SNR) ratio (7, 8). Another important benefit is the detection of cortical gray matter lesions (8). Because a significant proportion of surgical candidates has no relevant structural MRI abnormalities on 1.5T or 3.0T MRI, the introduction of 7 Tesla MRI techniques and diagnostic tools might provide a better surgical outcomes in these patients (4, 9).

Along with the increasing recognition of 7T MRI in patients with epilepsy, several studies have reviewed the clinical value of 7T MRI (7, 10, 11). However, no studies have quantified the diagnostic value of 7T MRI in comparison with conventional 1.5T or 3.0T MRI. A unifying evidence-based systematic summary of clinical studies might reveal the clinical value of 7T MRI. We performed a systematic review and meta-analysis of published clinical reports to determine the added value of 7T compared with the diagnostic performance of 1.5T or 3T MRI and to analyze the diagnostic value of 7T MRI in patients with epilepsy.

## Materials and Methods

### Article Search Strategy and Study Selection

**Figure 1:**
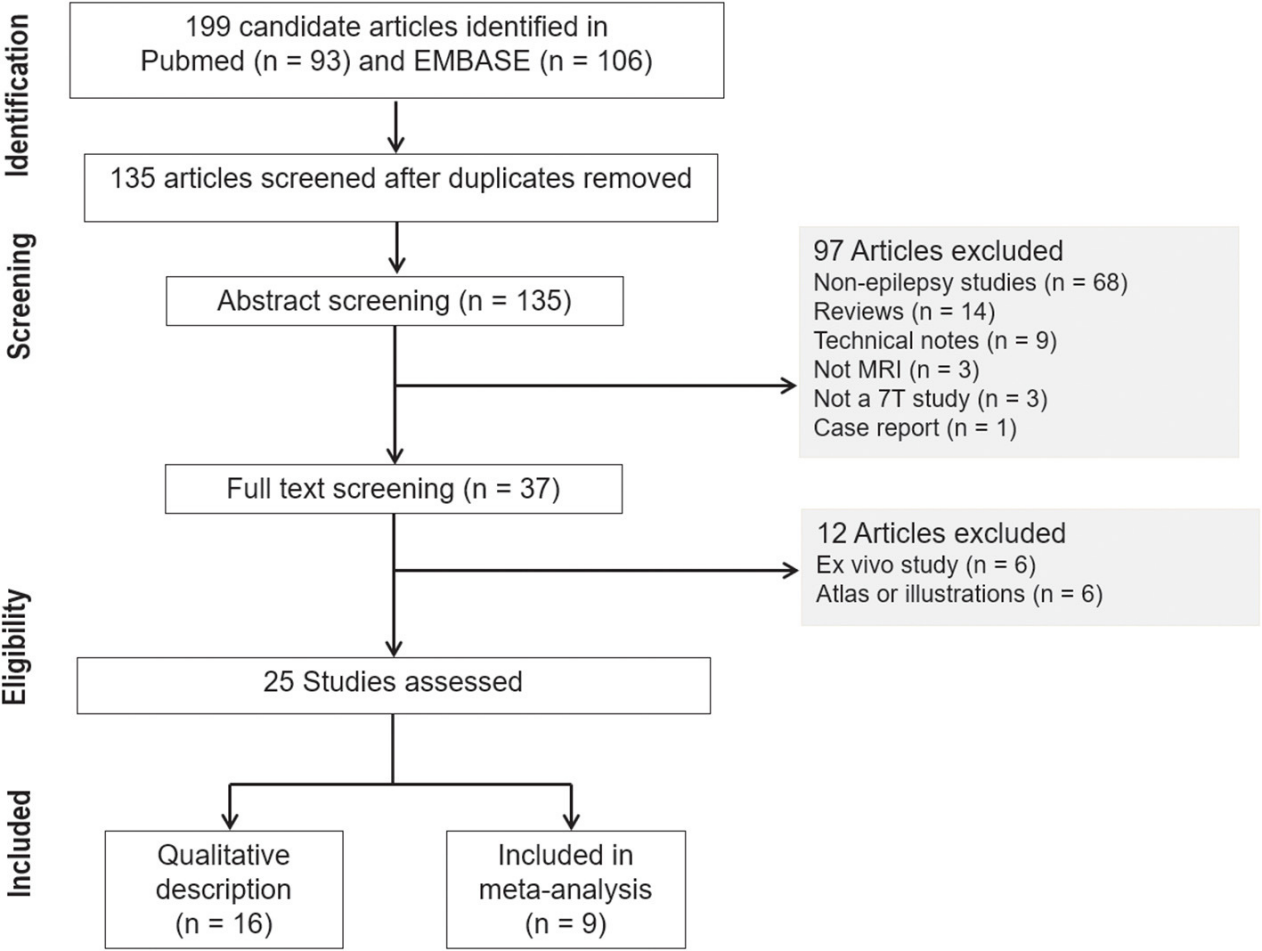
Flow diagram of the study selection process.

The systematic review and meta-analysis were conducted and reported in accordance with the Preferred Reporting Items for Systematic Reviews and Meta-Analysis–Diagnostic Test Accuracy (PRISMA-DTA) guidelines (12). The search terms used to find studies were “epilepsy” AND “magnetic resonance imaging” OR “MR imaging” AND “7T” OR “7Tesla” OR “7T” in the MEDLINE (National Center for Biotechnology Information) and EMBASE databases. The inclusion process is shown in Figure 1. From 199 identified articles, 135 remained after the removal of duplicates. Screening of the abstracts was performed, and 68 non-epilepsy studies, 14 review articles, 9 technical notes, 3 non-MRI studies, 3 non-7T studies, and 1 case report were excluded. Only articles published in English were reviewed. Full-text reviews of the remaining 37 potentially eligible articles were performed by two experienced reviewers working in consensus (J.E.P. and W.H.S., with 11 and 26 years of experience in radiology). In this process, 6 *ex-vivo* studies and 6 atlas or illustration articles were excluded. Finally, 25 articles were included in the main analysis, which consisted of 10 articles reporting a diagnostic performance study.

### Quality Assessment

Study quality was measured using the Newcastle Ottawa scale (13) for cohorts and modifications for case-control studies and case series that converged into eight items that could be categorized into three domains: selection, confounder, and outcome for cohort studies and selection, comparability, and exposure for case-control studies and case series.

### Data Extraction

All articles were reviewed by two independent board-certified radiologists and the following general data were extracted: first author, journal, year of publication, characteristics of patients with epilepsy, type of epilepsy, age, and sex at epilepsy onset. The age and sex of controls were recorded. MRI protocols and parameters were recorded as well as whether structural and functional MRI were performed. The study design was recorded as case series/case study, case control, or cohort study (14). When there were quantitative parameters, these values were extracted from the main text, tables, or figures. In studies that included healthy controls, the quantitative parameters of the healthy controls were collected separated.

Epilepsy type was followed by the International League Against Epilepsy (ILAE) classification of seizures and epilepsy (15). Epilepsy type included generalized epilepsy, focal epilepsy, combined generalized and focal epilepsy, and unknown. Then, epilepsy with structural etiology included mesial temporal lobe epilepsy (TLE) with hippocampal sclerosis, hypothalamic hamartoma, focal cortical dysplasia (FCD), polymicrogyria, and tuberous sclerosis, or acquired structural etiology including hypoxic-ischemic encephalopathy, stroke, trauma, or infection (15). Focal epilepsy included both lesional and non-lesional epilepsy, and epilepsy with clear structural etiologies were recorded as lesional.

### Statistical Analysis

In 9 studies reporting a detection rate, we performed a meta-analysis to determine the epileptogenic zone when comparing 7T with 1.5T/3.T MRI. The pooled detection rate for focal lesions in patients with epilepsy was obtained by the Hartung-Knapp adjustment for random-effects models (16). Heterogeneity was evaluated using the Higgins inconsistency index (*I*^2^) test and Cochran Q test (17), and *P* < 0.1 in the Q test and *I*^2^ values >50% were considered to indicate significant heterogeneity (18). Publication bias was assessed by a funnel plot and Begg test, and a *P* < 0.1 in the Begg test indicated significant publication bias. All statistical analyses were performed using the “meta” package in R software (version 3.6.0, R Foundation for Statistical Computing, Vienna, Austria) by an expert statistician (L.J.S. with 10 years of experience).

## Results

### Study Characteristics and Quality Assessment

Our search process is shown in Figure 1. The basic characteristics of the 25 articles included in this study are summarized in Table 1. The studies were published in 2011 (1 article), 2013 (1 article), 2015 (1 article), 2016 (6 articles), 2017 (5 articles), 2018 (5 articles), 2019 (4 articles), and 2020 (2 articles). There were 467 patients and the mean patient number was 18.9 (standard deviation, 12.5; range, 6–66), with a mean age of 30.1 years (standard deviation, 7) with 216 men except for 2 studies with no information regarding age and sex. There were 167 healthy controls with a mean age of 32.3 years (standard deviation, 7) with 75 men and 1 study with no information regarding age and sex. Ten studies included pediatric patients.

**Table 1:** Baseline characteristics of 25 studies with 7T MRI included in this analysis.

	**Author (year of publication)**	**Journal**	**Study design**	**Assessment**	**7 T MRI protocols**					**Patients with epilepsy**					**Control**		
					**Vendor**	**T1**	**T2/FLAIR**	T2*	**Others**	**Epilepsy type**	**Pediatric**	***N***	**Sex M:F**	**Age (year)**	***N***	**Sex M:F**	**Age (year)**
1	Henry et al. (19)	*Radiology*	Case-control	Qualitative/anithaQuantitative	Magnex, Oxford, England	3D MPRAGE	FSE	-	-	TLE (HS)	-	11	5:6	26	13	8:5	28
2	Pan et al. (39)	*Epilepsia*	Cohort	Quantitative	Varian,anitha Agilent Technologies, USA	IR-GRE	-	-	MRSI	Focal	-	25	7:18	33.9	-	-	-
3	De Ciantis et al. (26)	*AJNR Am. J. Neuroradiol*.	Case study, Add-on	Qualitative	Excite HDx, GE Healthcare, USA	3D FSPGR	T2 FSE, FLAIR	2D GRE 3D SWAN	2D FSE-IR	Polymicrogyria	-	10	4:6	30.1	-	-	-
4	Colon et al. (20)	*Acta Neurol. Belg*.	Case study, Add-on	Qualitative	Achieva, Philips, Netherlands	3D	T2 TSE, FLAIR	GRE	-	FCD*	-	11	7:4	37.3	-	-	-
5	De Ciantis et al. (27)	*Epilepsia*	Case study, Add-on	Qualitative	Discovery 950 MRI, GE Healthcare, USA	3D FSPGR	T2 FSE, 3D FLAIR	2D GRE 3D SWAN	2D FSE-IR	Focal	Yes	21	12:9	24.2	-	-	-
6	Grouiller et al. (42)	*Magma*	Case study, Add-on	Qualitative	Magnetom, Siemens, Germany	3D MP2RAGE	-	2D SWI	EEG-fMRI	Focal, lesional	Yes	9	7:2	25.9	-	-	-
7	Kwan et al. (28)	*J. Neurol. Sci*.	Case study, Add-on	Qualitative	Agilent, Siemens, Germany	3D MPRAGE	-	SWI	-	TLE	-	13	7:6	31.5	-	-	-
8	Springer et al. (21)	*Invest. Radiol*.	Case-control, Add-on	Qualitative/anithaQuantitative	Magnetom, Siemens, Germany	MP2RAGE	T2 TSE, FLAIR TSE	2D GRE	DWI	Focal Lesional or non-lesional	-	9	Unknown	Unknown	10	6:4	27.5
9	Veersema et al. (29)	*Epileptic Discorder*.	Case series	Qualitative	Philips, OH, USA	3D	3D	2D GRE	3D DIR	FCD	Yes	6	2:4	21	-	-	-
10	O'Hallloran et al. (43)	*Neuroreport*	Case-control	Quantitative	Unknown	3D MP2RAGE	-	-	DTI	Focal, non-lesional	-	8	4:4	33	8	6:2	39
11	Santyr et al. (30)	*J. Mag. Reson. Imaging*	Case-control	Qualitative/anithaQuantitative	Agilent, Siemens, Germany	3D MPRAGE	-	-	-	TLE	-	13	7:6	33.5	20	10:10	31.2
12	Stefanits et al. (31)	*Invest. Radiol*.	Case series	Qualitative	Magnetom, Siemens, Germany	-	T2 FSE	-	-	TLE	-	13	8:5	38.7	-	-	-
13	Veersema et al. (30)	*Epilepsia Open*	Case study, Add-on	Qualitative	Achieva, Philips, Netherlands	3D MPRAGE	3D T2, 3D FLAIR	GRE		Focal, Lesional or non-lesional	-	40	Unknown	18	-	-	-
14	Voets et al. (32)	*Sci. Rep*.	Case-control	Quantitative	Siemens, Germany	3D MPRAGE	-	SWI	MRS	TLE	-	12	7:5	35.2	12	3:9	29.5
15	Feldman et al. (41)	*Seizure*	Case-control	Quantitative	Magnetom, Siemens, Germany	-	T2 TSE	-	-	Focal, Lesional, MRI-negative	-	21	13: 8	33	17	11:6	33
16	Obusez et al. (22)	*Neuroimage*	Case study, Add-on	Qualitative	Magnetom, Siemens, Germany	3D MP2RAGE	2D TSE	3D SWI	-	Focal, Lesional	Yes	55	36:19	31.2	-	-	-
17	Pittau et al. (23)	*J. Neuroimaging*	Case series	Qualitative	Magnetom, Siemens, Germany	3D MP2RAGE	FLAIR	3D SWI	DTI	Focal, Lesional	-	7	4:3	24.8	-	-	-
18	Rutland et al. (38)	*Seizure*	Case-control	Quantitative	Magnetom, Siemens, Germany	3D MP2RAGE	T2 TSE	-	DTI	Focal, MRI-negative	-	25	9:16	31.2	25	9: 16	31.7
19	Sun et al. (24)	*Neuroradiology*	Case study, Add-on	Qualitative	Magnetom, Siemens, Germany	3D MPRAGE	2D T2 TSE, 3D FLAIR	3D SWI	-	Tuberous sclerosis	Yes	10	5:5	13.2	-	-	-
20	Bartolini et al. (33)	*AJNR Am. J. Neuroradiol*.	Case study, Add-on	Qualitative	Discovery 950 MRI, GE Healthcare, USA	3D MPRAGE	2D T2 FSE, 3D FLAIR	3D SWAN, 2D GRE	-	FCD	Yes	12	7:5	23.3	-	-	-
21	Feldman et al. (34)	*PLoS ONE*	Case-control, Add-on	Qualitative	Magnetom, Siemens, Germany	3D MPRAGE, MP2RAGE	2D T2 TSE, 3D FLAIR	2D SWI	-	Focal, Lesional	Yes	37	20:17	36.1	21	15:6	34
22	Shah et al. (36)	*Hum. Brain Mapp*.	Case-control	Quantitative	Magnetom, Siemens, Germany	3D MPRAGE	T2 TSE	-	BOLD fMRI	Focal	Yes	13	3:10	45.4	24	Unknown	Unknown
23	Zhang et al. (25)	*Seizure*	Case study, Add-on	Qualitative	Unknown, Siemens, Germany	3D MPRAGE	T2 FSE	-	-	TLE	Yes	39	20: 19	27.0	-	-	-
24	Feldman et al. (40)	*Epilepsia*	Case-control	Quantitative	Magnetom, Siemens, Germany	3D MPRAGE	-	3D SWI		Focal, MRI-negative	-	34	14:20	37	17	7:10	37
25	Lampinen et al. (37)	*Epilepsia*	Case series	Quantitative	Achieva, Philips, Netherlands	3D TFE	3D FLAIR	-	DTI	FCD	Yes	13	8:5	32	-	-	-

*FSPGR, fast-spoiled gradient echo; MPRAGE, magnetization prepared rapid gradient echo; MP2RAGE, variation of magnetization prepared rapid gradient echo; TLE, Temporal lobe epilepsy; FCD, Focal cortical dysplasia; yr, year; N = number*.

Structural MRI included high-resolution T1, T2, and susceptibility-weighted imaging and functional MRI included diffusion tensor imaging (*n* = 4), functional MRI (*n* = 2), and MR spectroscopy (*n* = 2). Study design included 10 case studies, 10 case-control studies, 4 case series, and 1 cohort study.

Regarding epilepsy type, all studies included focal or combined generalized and focal epilepsy (25/25 studies). Of these, four studies included patients with focal but non-lesional epilepsy (MRI-negative) on 1.5T or 3.0T. Twelve studies (12/25, 48%) specified the etiology; of these, 6 included TLE (6/25, 24%), 4 included FCD (4/25, 16%), 1 study included polymicrogyria, and 1 study included tuberous sclerosis.

Study quality was measured with the Newcastle Ottawa scale ranging from 1 to 6 (Supplementary Data). Most studies lost points as they did not include independent validation in addition to an unclear non-response rate.

### Systematic Review for Qualitative Assessment Using Structural MRI on 7T

A summary of studies according to the study endpoint, studies for meta-analysis, reliability or descriptive analyses, and potential imaging biomarkers is shown in Table 2. Qualitative assessment of morphologic characteristics on structural MRI protocols was performed for 16 studies (64%), of which a scoring system was assessed in 7 studies and the detection rate was assessed in 9 studies. In all studies with a scoring system (19–25), 7T MRI scored higher than 3T MRI for lesion conspicuity of morphologic characteristics and/or reader's confidence (21). The inter-rater agreement of the diagnostic confidence scale was higher on 7T (92.3%) than on 3T (57.7%) (21).

**Table 2:** Summary of 7T Epilepsy Study on 7T according to study endpoint.

**No**	**Author (year of publication)**	**Journal**	**Study design**	**Assessment**	**Qualitative/Semi-qualitative**				**Quantitative**		
	**Study endpoint**				**Measurement**	**No. of readers**	**Measurement**	**Result**	**Measurement**	**Patients**	**Healthy control**
**DETECTION RATE (META-ANALYSIS)**											
3	De Ciantis et al. (26)	*AJNR Am. J. Neuroradiol*.	Case study, Add-on	Qualitative	Detection	3 in consensus	Count	4/6 new bilateral detection	**-**	**-**	**-**
5	De Ciantis et al. (27)	*Epilepsia*	Case study, Add-on	Qualitative	Detection	3 in consensus	Count	6/21 new detection	**-**	**-**	**-**
7	Kwan et al. (28)	*J. Neurol. Sci*.	Case study, Add-on	Qualitative	Detection	1	Count	13/13 (7T) 10/13 (1.5 or 3.0T)	**-**	**-**	**-**
9	Veersema et al. (29)	*Epileptic Discorder*.	Case series	Qualitative	Detection	Unknown	Count	4/6 (7T) 2/6 (3.0T)	**-**	**-**	**-**
11	Santyr et al. (30)	*J. Mag. Reson. Imaging*	Case-control	Qualitative/anithaQuantitative	Detection, Morphologic abnormalities	2	4-point scale	6/9 new detection	Hippocampal volumetry	Per subregion	Per subregion
12	Stefanits et al. (31)	*Invest. Radiol*.	Case series	Qualitative	Morphologic abnormalities	4	Binomial rating	8/13 detection no 3.0T	**-**	**-**	**-**
13	Veersema et al. (32)	*Epilepsia Open*	Case study, Add-on	Qualitative	Detection	1	Count	9/40 new detection	**-**	**-**	**-**
20	Bartolini et al. (33)	*AJNR Am. J. Neuroradiol*.	Case study, Add-on	Qualitative	Detection	2 in consensus	Count	10/12 detection (7T) vs. 9/12 detection (3T)	**-**	**-**	**-**
21	Feldman et al. (34)	*PLoS ONE*	Case-control, Add-on	Qualitative	Detection	2 in consensus	Count	25/37 new detection	**-**	**-**	**-**
**DESCRIPTIVE OR RELIABILITY STUDY**											
1	Henry et al. (19)	*Radiology*	Case-control	Qualitative/anithaQuantitative	Hippocampal digitations	2	Likert scale	0.93 (intrarater)/0.80 (interrater)	Subregional volume	(ictal, CA1-3) 130 mm^3^	(CA1-3) 238^3^ (CA4- dentate) 172mm^3^
4	Colon et al. (20)	*Acta Neurol. Belg*.	Case study, Add-on	Qualitative	7x2 morphologic features	3	Likert scale	Superior 7 out of 14 items	**-**	**-**	**-**
8	Springer et al. (21)	*Invest. Radiol*.	Case-control, Add-on	Qualitative/anithaQuantitative	Morphologic abnormalities	2	Diagnostic confidence scale (10)	Interrater 92.3 % (7T)/57.7% (3T)	CNR, SNR	Per modality	Per modality
**DETECTION RATE (META-ANALYSIS)**											
16	Obusez et al. (22)	*Neuroimage*	Case study, Add-on	Qualitative	Lesion conspicuity	10	5-point scale	More conspicuous on 7.0 T than 3.0T	**-**	**-**	**-**
17	Pittau et al. (23)	*J. Neuroimaging*	Case series	Qualitative	Lesion conspicuity	Unknown	Descriptive	More conspicuous on 7.0 T than 3.0T	**-**	**-**	**-**
19	Sun et al. (24)	*Neuroradiology*	Case study, Add-on	Qualitative	Lesion conspicuity	3 in consensus	Descriptive	More conspicuous on 7.0 T than 3.0T			
23	Zhang et al. (25)	*Seizure*	Case study, Add-on	Qualitative	Morphologic abnormalities	1	4-rating system	Higher score on 7T than 3T	**-**	**-**	**-**
**POTENTIAL IMAGING BIOMARKERS**											
2	Pan et al. (39)	*Epilepsia*	Cohort	Quantitative	MRSI NAA/Cr > cutoff	1	Count of voxels	PPV 100%, NPV 73%, Sens 82%, Spec 100%	NAA/Cr	1.32 ± 0.10	1.21 ± 0.13
6	Grouiller et al. (42)	*Magma*	Case study, Add-on	Qualitative	Mapping of eloquent cortex	Unknown	Voxels	Same with 1.5T or 3.0T	**-**	**-**	**-**
10	O'Hallloran et al. (43)	*Neuroreport*	Case-control	Quantitative	**-**	**-**	**-**	**-**	U-fiber count	1,500	1,700
14	Voets et al. (35)	*Sci. Rep*.	Case-control	Quantitative	**-**	**-**	**-**	**-**	Hippocampal volumetry, metabolites (glutamate)	Per subregion	Per subregion
15	Feldman et al. (41)	*Seizure*	Case-control	Quantitative	**-**	**-**	**-**	**-**	Perivascular space, count	1,603	1,902
18	Rutland et al. (38)	*Seizure*	Case-control	Quantitative	**-**	**-**	**-**	**-**	Fiber density	Per subregion	Per subregion
22	Shah et al. (36)	*Hum. Brain Mapp*.	Case-control	Quantitative	**-**	**-**	**-**	**-**	Hippocampal volumetry, connectivity	Per subregion	Per subregion
24	Feldman et al. (40)	*Epilepsia*	Case-control	Quantitative	**-**	**-**	**-**	**-**	Vessel density (cm^3^)	2.1–2.4	2.7
25	Lampinen et al. (37)	*Epilepsia*	Case series	Quantitative	**-**	**-**	**-**	**-**	Diffusion parameter (FA, MK)	Per disease	Per disease

*HIA, hippocampal asymmetry score; N, number; FA, fractional anisotropy; MK, mean kurtosis; CNR, contrast-to-noise ratio; SNR, signal-to-noise ratio; NAA, N-acetylacetate*.

The lesion conspicuity was higher on descriptive studies of 7T using high-resolution T1-weighted imaging of magnetization-prepared rapid gradient echo (MPRAGE) and SWI. The signs of TLE include abnormal hippocampal digitations, size differences (atrophy), abnormality of shape, and increased signal (19).

The signs of FCD include increased cortical thickness, cortical thinning, abnormal sulcation, regional hypoplasia/atrophy, transmantle sign, blurring of the gray/white matter junction, T2-weighted hyperintensity, and T1-weighted hypointensity in subcortical white matter. The diagnostic confidence was superior in 7 of 14 items on 7T compared with 3T (20).

For tuberous sclerosis (24), 7T detected tuber extension beyond the margins identified on conventional 3 T MR images, which offered a better delineation of cortical abnormalities.

### Meta-Analysis of the Detection Rate of 7T Compared With 3T

**Figure 2:**
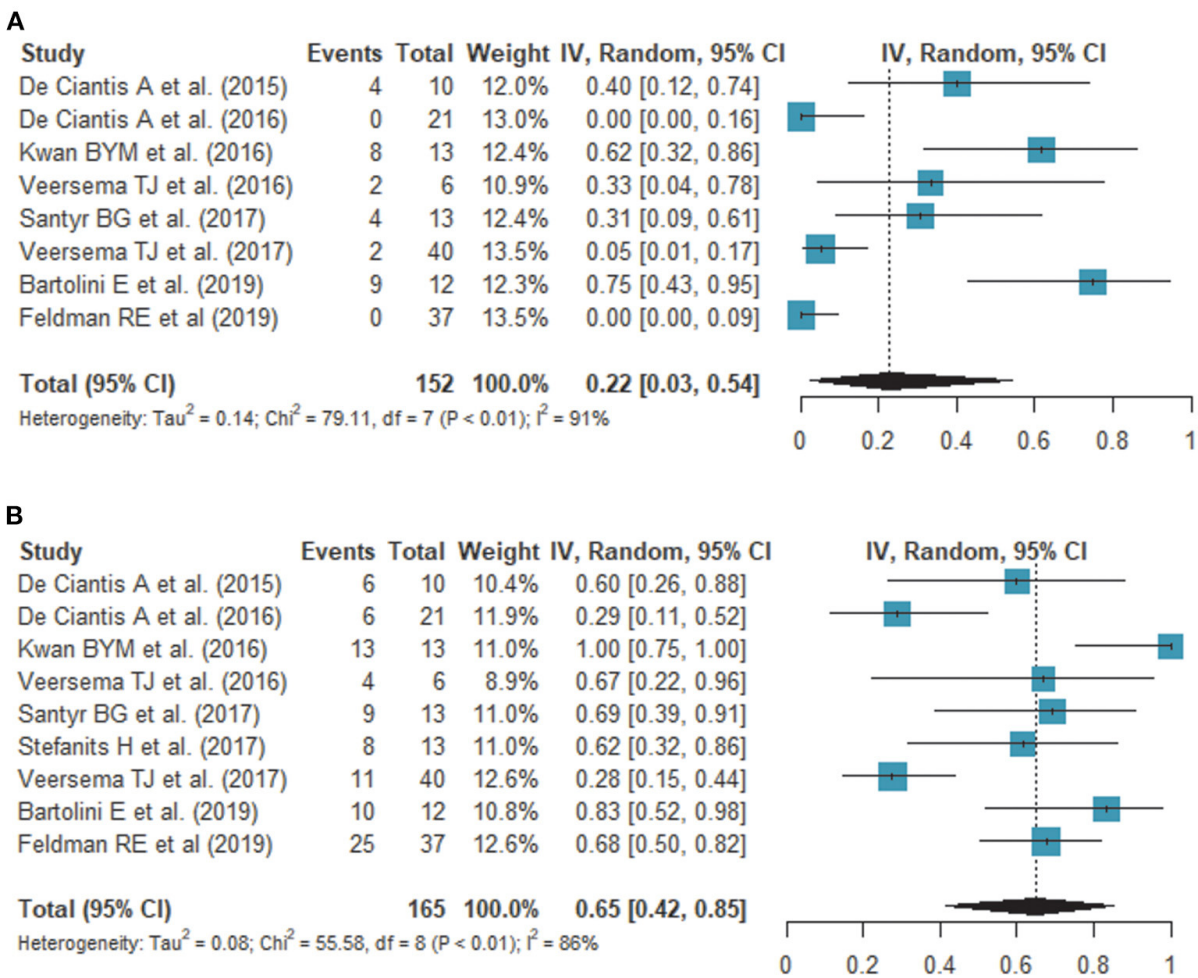
Forest plots show the comparison of the detection rate on 3.0T **(A)** and 7.0T **(B)** MRI. Overall detection rates of 7T and 3T MRI are 65 and 22%, respectively, showing a statistically significant difference.

Nine studies assessed the detection rate of lesional epilepsy (26–34), of which 9 studies (160 patients) reported a detection rate on 7T and 8 studies (152 patients) reported a detection rate on 3T (Table 2). According to the meta-analysis of 9 studies, the overall detection rate of 7T MRI was 65% (95% confidence interval [CI]: 42 %, 85%) (Figure 2A) while that of 3T MRI was 22% (95% CI: 3%, 54%) (Figure 2B). The analysis revealed heterogeneity for 7T (*I*^2^ = 85.6%) and 3T (*I*^2^ = 91.2%). A funnel plot revealed no publication bias in data of both 7T and 3T (both *P* > 0.01, Begg test) (Supplementary Figure).

The overall detection rate was 33% higher by 7T compared with 3T. There was new bilateral detection of polymicrogyria (26), as well as new detections of FCD (27, 29, 32, 33) and hippocampal abnormalities (28, 30, 31). Of note, susceptibility weighted imaging (SWI) demonstrated leptomeningeal venous abnormalities (29) or intracortical black line signs (33), which is associated with overlying focal cortical dysplasia. One study showed a histopathologic correlation between a negative finding on 7T and an epileptogenic lesion (gliosis) (27).

### Systematic Review of Quantitative Assessment and Potential Imaging Biomarkers on 7T

Quantitative assessment for potential imaging biomarkers was available for 10 studies (10/25, 40%) including 4 studies with hippocampal volumetry (19, 30, 35, 36), 3 studies with diffusion tensor parameters (37) or fiber tracking (38, 43) on DTI, 2 studies with metabolites from MR spectroscopy (35, 39), 1 study with vessel density (40), 1 study with perivascular space (41), and 1 study with functional MRI (42).

Hippocampal volumetry was quantitatively measured in hippocampal subfields in all volumetric studies (19, 30, 35, 36), and identified selectively greater ipsilateral Ammon horn atrophy (19) and CA1 and CA4+dentate gyrus atrophy (30) in patients with TLE. High-angular-resolved diffusion-weighted imaging revealed lower u-fiber counts in subjects with epilepsy (43) compared with healthy controls. Hippocampal tractography demonstrated patients with left temporal seizure focus (38), exhibited increased connectivity of certain ipsilateral subfields, especially the subiculum, presubiculum, and parasubiculum, and reduced connectivity for contralateral subfields, such as CA1 and subiculum.

With improved contrast and resolution, the depiction of the perivascular space was significantly improved on 7T using axial T2-weighted TSE sequences (41). In a case control study of 21 epilepsy patients and 17 healthy controls (41), the distribution of the perivascular space was significantly more asymmetric in epilepsy patients, and the region of maximum asymmetry was within the suspected seizure onset zone in 72% of cases. Similar results were reported in a vessel density study using SWI (40) where vessel density was highly symmetric in the hippocampus in controls, whereas the mean vessel density asymmetry was greater in neocortical and MTL epilepsy patients, where the decrease in vessel density was ipsilateral to the suspected seizure onset zone.

The N-acetyl aspartate (NAA)/ Creatinine (Cr) ratio (39) and glutamate (35) on MR spectroscopic imaging (MRSI) are potential biomarkers in epilepsy. Using voxels exhibiting a decreased NAA/Cr ratio, neocortical abnormalities were detected and localized surgery was possible (35): in a study of 25 patients, the positive predictive value for the concordance of MRSI with a good outcome was 100%, the negative predictive value was 73%, with a sensitivity of 82%, and a specificity of 100%. In another study using single-voxel MR spectroscopy (35), 7 out of 8 patients had altered metabolite concentrations of glutamate, glutamine, myo-inositol, NNN, Cr, and choline, as well as markedly reduced glutamine levels of 62.5% compared with that of healthy controls.

## Discussion

Our findings support the use of 7T MRI in patients with epilepsy to identify morphologic abnormalities and define subregions of anatomic structures for surgical guidance by providing a higher signal-to-noise ratio, improved image uniformity, and better spatial resolution. Structural lesions should be identified on MRI for the presurgical workup, and the epileptogenic focus should be localized for good surgical outcomes (1, 2, 4). Moreover, the potentially epileptogenic focus should be depicted to obtain target areas for subsequent electrode implantation or eliminate the need for intracranial EEG monitoring (27), thereby facilitating surgery. In patients with epilepsy, 7T MRI detected or better characterized lesions and was a more focused and less invasive approach.

Lesions of an unknown etiology are better delineated on 7T MRI. The internal structure and extent of lesions are best visible on T2- and T2^*^-weighted sequences in patients with FCD and hippocampal sclerosis. Particularly, the flag-like three-layer appearance with the middle hypointense line on T2-weighted images (20) in patients with FCD facilitates the visual diagnosis (20, 33). In a study involving patients with polymicrogyria, 7T MRI revealed more extensive areas and occasionally detected bilateral disease involvement (26) in patients previously assessed to have unilateral disease involvement. Moreover, high magnetic susceptibility properties improve detection of small cavernous cortical hemangioma (20) and vascular hippocampal abnormalities with the T2^*^-weighted sequence (40).

This meta-analysis provides quantitative summary estimates of the detection rates in clinical studies on epilepsy. Previous review articles reported that the detection of lesions in patients with epilepsy can be improved by ~23–30% (8) on 7T, but that the results were inconsistent and had not been reviewed systemically. In this meta-analysis, the pooled detection rate of 7T MRI was higher than that of 3T MRI, with overall detection rates of 65% and 22%, respectively, showing a 33% difference. Owing to a high SNR, 7T MRI offers better spatial resolution, enabling better depiction of anatomical structures. Anatomic alterations include the transmantle sign in FCD (33), irregular outer surface and irregular cortical-white matter junction in polymicrogyria (26), and disruption of the internal architecture of the hippocampus in hippocampal sclerosis (19). These findings result from a thin slice section, small voxel size, and high magnetic susceptibility of 7T MRI.

Recommended specific sequences and paradigms as potential imaging biomarkers for epilepsy on 7T MRI are the improved conventional T1/T2-weighted sequence, diffusion tensor imaging, the T2^*^-weighted sequence/SWI, and MR spectroscopy. Diffusion tensor-based tractography provides detailed white matter architecture and better quantification of fiber density (38, 43) and connectivity (36). T2^*^-weighted and SWI sequences better demonstrate cavernomas (44) and enlarged perivascular structures, indicating adjacent white matter disease (45). MR spectroscopy on 7T MRI provides high spectral resolution of molecules, thereby enabling the quantification of numerous metabolites not visible on 3.0T or 1.5T MRI (46). In addition to NAA, glutamine and glutamate (35) are metabolites that may be used to detect neurochemical abnormalities in regions exhibiting structurally normal morphology.

Our study had some limitations. First, although studies had paired data for both 3 T/1.5T and 7T MRI, comparisons of paired proportions were unavailable because articles included the detection rate but not the false-positive and false-negative, or true-negative rate in a 2 × 2 table to compare the statistical significance. Second, most of studies included a small number of patients (case study/case series) and showed heterogeneity with a low level of evidence. A prospective cohort study, with available surgical outcomes and diagnostic performance is warranted to achieve a higher level of evidence. As for the future perspective, a meta-analysis of more formalized clinical trials and larger studies would provide the clinical context and outcomes necessary for 7T MRI to become a valid clinical tool.

In conclusion, 7T MRI has value in delineating morphologic abnormalities with higher detection rate compared with clinical MRI. Most studies were conducted as case series or case studies, and a cohort study design with clinical outcomes is necessary.

## Data Availability Statement

The original contributions presented in the study are included in the article/Supplementary Material, further inquiries can be directed to the corresponding authors.

## Author Contributions

JP and E-NC contributed to the conception of the study, analysis, and manuscript preparation. DJ revised manuscript. All authors helped to perform the analysis with constructive discussions.

## Conflict of Interest

The authors declare that the research was conducted in the absence of any commercial or financial relationships that could be construed as a potential conflict of interest.

## Abbreviations

MRI: magnetic resonance imaging
SNR: signal-to-noise
PRISMA-DTA: Preferred Reporting Items for Systematic Reviews and Meta-Analysis–Diagnostic Test Accuracy.
