# Supplementary material for: Utility of 7 Tesla Magnetic Resonance Imaging in Patients With Epilepsy: A Systematic Review and Meta-Analysis
Source: Front Neurol. 2021 Mar 19;12:621936. doi: 10.3389/fneur.2021.621936 (PMC8017213; doi:10.3389/fneur.2021.621936)
Supplement: Supplementary file 1 [file Data_Sheet_1.docx]

**
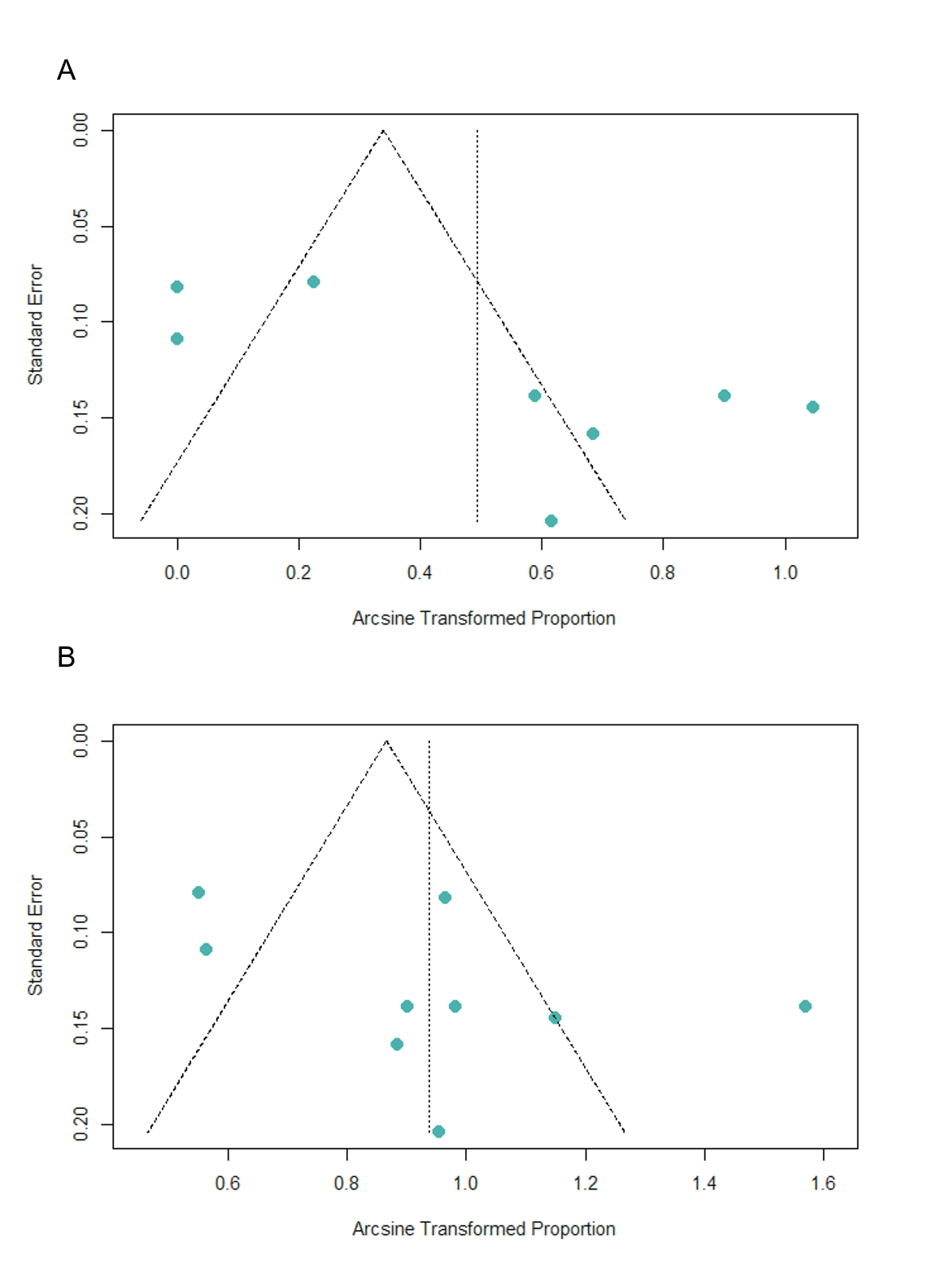
**

**Supplementary Figure.** Funnel plot shows the clinical studies included in the meta-analysis. Substantial asymmetry is indicative of potential publication bias within the pool of identified studies. The funnel plot revealed there was no publication bias in data for both 7T and 3T.
